# How to evaluate performance of prediction methods? Measures and their interpretation in variation effect analysis

**DOI:** 10.1186/1471-2164-13-S4-S2

**Published:** 2012-06-18

**Authors:** Mauno Vihinen

**Affiliations:** 1 Institute of Biomedical Technology, FI-33014 University of Tampere, Finland; 2 BioMediTech, Tampere, Finland; 3 Department of Experimental Medical Science, Lund University, SE-221 84 Lund, Sweden

## Abstract

**Background:**

Prediction methods are increasingly used in biosciences to forecast diverse features and characteristics. Binary two-state classifiers are the most common applications. They are usually based on machine learning approaches. For the end user it is often problematic to evaluate the true performance and applicability of computational tools as some knowledge about computer science and statistics would be needed.

**Results:**

Instructions are given on how to interpret and compare method evaluation results. For systematic method performance analysis is needed established benchmark datasets which contain cases with known outcome, and suitable evaluation measures. The criteria for benchmark datasets are discussed along with their implementation in VariBench, benchmark database for variations. There is no single measure that alone could describe all the aspects of method performance. Predictions of genetic variation effects on DNA, RNA and protein level are important as information about variants can be produced much faster than their disease relevance can be experimentally verified. Therefore numerous prediction tools have been developed, however, systematic analyses of their performance and comparison have just started to emerge.

**Conclusions:**

The end users of prediction tools should be able to understand how evaluation is done and how to interpret the results. Six main performance evaluation measures are introduced. These include sensitivity, specificity, positive predictive value, negative predictive value, accuracy and Matthews correlation coefficient. Together with receiver operating characteristics (ROC) analysis they provide a good picture about the performance of methods and allow their objective and quantitative comparison. A checklist of items to look at is provided. Comparisons of methods for missense variant tolerance, protein stability changes due to amino acid substitutions, and effects of variations on mRNA splicing are presented.

## Background

Gene and genome sequencing speed is ever increasing and thus lots of genetic variation information is available. The technological development of sequencing methods has led to a situation where the interpretation of the generated data is a severe bottleneck for the use of genetic information. Numerous prediction methods have been developed during the last decade to address the relevance of gene and protein variants to pathogenicity. General tolerance methods predict whether the variants are disease-related or not (or affect protein function or not), and specific methods are used to address variation effect mechanisms [1]. These methods can be useful. However, until recently their true applicability and performance have not been studied systematically [2-5]. When methods are originally published, authors provide some performance measures, which are usually not comparable with other methods due to the use of different training and test datasets, different reported measures etc. The scope of this article is to discuss how the assessment of method performance should be done and interpretation of the results and the choice of the best methods. The text is mainly intended for scientists who are users of predictors without training in statistics or computer science. Method developers are taken into account by providing a checklist of items to be reported with methods. The examples discussed are related to prediction of variant effects, but description of methods and evaluation measures is general and thereby not application domain specific.

### Method testing schemes

**Figure 1:**
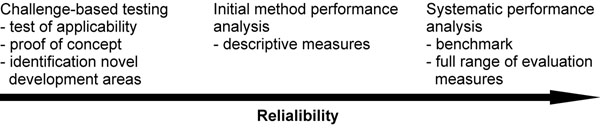
**Method performance analysis schemes** The performance of the computational methods can be addressed with three different approaches which yield different reliability for the assessment.

Three approaches can be used for testing method performance and can be classified according to increasing reliability (Fig. 1).

Challenges aim to test whether certain problems can be addressed with existing tools and to find out what kind of methods will be needed in the future. Critical Assessment of Structure Predictions (CASP) [6] was the first challenge of this kind in biosciences. The idea was, and still is, even when CASP has been running for 20 years, to test how prediction methods behave on different protein structure related tasks. The method developers apply their systems without knowing the correct result (blind test), which however is available for the challenge assessors. This setup allows independent testing of method performance. In a similar vein, other critical assessment challenges have been organized e.g. for Critical Assessment of protein Function Annotation (CAFA) [7] and Critical Assessment of PRediction of Interactions (CAPRI) [8].

CAGI, Critical Assessment of Genome Interpretation (http://genomeinterpretation.org/), is a challenge for method developers in the field of phenotypic impacts of genomic variation. The second CAGI prediction season was organized during fall 2011. These challenges do not aim for systematic analysis of predictions, instead they assess what is currently doable, providing proof of concept, charting where to direct future efforts, and identifying new areas where predictive approaches would be needed.

The second test strategy is typically used by method developers to test their approaches. These are usually done with developer collected test sets (especially when benchmark datasets are lacking) and report certain performance parameters. Most often the testing is not comprehensive, and the results are incomparable with those obtained from other methods e.g. due to using different test sets. Sometimes evaluation parameters are selectively presented which leads to problems in determining the true merits and pitfalls of methods.

The third approach, systematic analysis, uses approved and widely accepted benchmark dataset(s) and suitable evaluation measures to explain method performance. It is hoped that in the future the variation effect program developers would use benchmark test sets and comparable measures. This is already the general practice e.g. in the multiple sequence alignment (MSA) field.

## Prediction methods for classification

A plethora of pattern recognition methods have been applied to problems in bioinformatics including rule based, statistical methods and machine learning -based methodologies. The goal of machine learning is to train a computer system to distinguish i.e. classify cases based on known examples. Machine learning methods include several widely differing approaches such as support vector machines, neural networks, Bayesian classifiers, random forests and decision trees.

In the following discussion we concentrate on machine learning methods as they are nowadays widely used to tackle complex phenomena, which would be otherwise difficult to handle. Successful machine learning method development requires good quality training set. The dataset should represent the space of possible cases. This space is huge for genetic variations as they can have so many different effects and underlying mechanisms. Another aspect is the choice of the machine learning approach. There is not a superior architecture among them. Third, the quality of the predictor depends on how the training has been done, which features are used to explain the phenomenon and optimization of the method.

**Figure 2:**
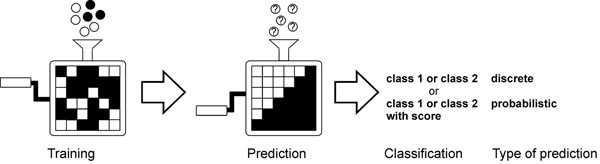
**Principles of machine learning** Machine learning is a form of supervised learning in which a computer system learns from given positive and negative instances to distinguish between cases belonging to the two classes. During training, positive and negative cases (black and white balls) are provided for the system, which leads to organization of the predictor (indicate by the arrangement of the black and white squares inside the predictor) such that it learns to separate the cases and thus can classify unknown cases (balls with question marks). Depending on the classifier, whether it yields in addition to the classification also a score for the prediction, the results can be called as discrete or probabilistic.

Fig. 2 depicts the principle underlying machine learning in a two-class classification task. The predictor is trained with known positive and negative instances in an approach called supervised learning. This leads to reorganization of the system, details of which differ according to the architecture employed. Once the method has learned to distinguish between the cases it can be applied to predict the class of unknown cases. The predictors can be classified as discrete or probabilistic depending on whether they provide a score, not necessarily a p value, for predictions. In the case of methods with discrete output, more or less *ad hoc* thresholds have been used to detect the most reliable events. Many machine learning based predictors are binary classifiers, however, it is possible to have more than two outputs e.g. by using multi-tier two-class prediction system.

Features describe the characteristics of the investigated phenomenon. If several features are available it is important to choose those, which best capture the phenomenon. This is partly due to the curse of dimensionality, which means that much more data are needed when the number of features increases. The volume of the feature space grows exponentially with the dimensionality such that the data become sparse and insufficient to adequately describe the pattern in the feature space. Another problem is overfitting, which means that the learner, due to sparse data, complex model or excessive learning procedure, describes noise or random features in the training dataset, instead of the real phenomenon. It is crucial to avoid overfitting as it leads to decreased performance on real cases.

Many predictors provide a measure for the probability of prediction, in this domain a measure of how likely the variation is pathogenic. This information can be used for ranking the investigated cases. A more advanced version is to obtain e.g. by bootstrapping an estimate of the standard error of the prediction indicative of the prediction reliability.

Many types of biological data are limited in quantity. The same data cannot be used both for method training and testing. The trick is to partition the dataset. This can be done in different ways, with cross-validation probably being the most popular of these. The dataset is divided into *k* disjoint partitions, one of which is used for testing and the others for training. This is repeated *k* times until all the partitions have been used as test set. Ten partitions i.e. ten times cross validation is the most common partitioning scheme. The average performance measures computed from the splits are used to describe the overall prediction performance. Random sampling is another approach, however, a problem is that the same cases may appear more than once in the test set and others not at all. Another computationally intensive validation approach is leave one out validation, an extreme case of cross validation with partitioning to the total number of instances. As the name implies, one case at time is left for validation while the remaining cases are used for training. The computational requirements may be prohibitive with large datasets. A problem especially for the last scheme is if there are some very similar cases in the dataset.

Typically the training set should contain about equal amount of cases in each class. Imbalance in the numbers of cases in the classes can cause problems during performance evaluation as discussed below. There are some ways to handle class imbalance.

## Principles of method evaluation

To test and compare predictors two requirements have to be met. There has to be available test dataset with known outcome and there has to be in place suitable prediction performance evaluation measures. Benchmark is a gold standard dataset - cases with experimentally validated known effects which represent the real world. These can be used for training machine learning methods as well as for testing the developed methods. The same data should however never be used for training and testing as that would only indicate the capacity of the method to memorize examples, not its generalization potential – how well it performs on instances outside the training set. High quality benchmark datasets require meticulous data collection often from diverse sources and careful checking of the correctness of the data.

Numerous measures have been developed to describe predictor performance, but no single measure captures all aspects of predictor performance. The measures mainly used, and how to interpret them will be discussed. Typically prediction methods are used as classifiers to define whether a case has the investigated feature or not. Results of this kind of binary predictor can be presented in a 2x2 confusion table also called contingency table or matrix. This, at first glance may appear simple to interpret, but the contrary is the case, as various composite aspects have to be jointly taken into account.

### Benchmark criteria

Benchmark can be defined as a standard or reference for evaluation, in this case prediction method performance. Benchmarks are widely used in computer science and technology. For example computer processor performance is tested with standardized benchmark methods. In bioinformatics there are benchmarks e.g. for multiple sequence alignment methods already 1990’s [9]. Novel MSA construction methods are routinely tested with alignment benchmarks such as BAliBASE [10] HOMSTRAD [11], OxBench suite [12], PREFAB [13], and SABmark [14] . Other bioinformatic benchmarks include protein 3D structure prediction [15-17], protein structure and function prediction [18], protein-protein docking [19] and gene expression analysis [20,21] benchmarks etc.

Benchmark usage varies between different communities. For variation effect predictions, benchmarks have not been available and thus authors have used different datasets. The situation has changed only recently with the release of VariBench (http://bioinf.uta.fi/VariBench/) (Nair and Vihinen, submitted).

**Figure 3:**
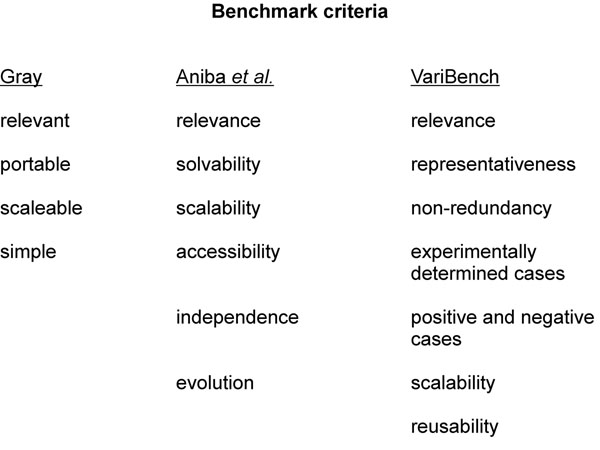
**Benchmark criteria** Criteria for benchmarks in three different studies. VariBench is the database specifically designed for variation benchmark datasets.

To be useful a benchmark should fulfill certain criteria. These criteria vary somewhat between the domains, but there are also some common features (Fig. 3). The criteria laid by Gray originally for database systems and transaction processing systems are still valid [22]. Criteria for MSA [23] and variation data (Nair and Vihinen, submitted) benchmarks have been defined. These include relevance, which means that the data have to capture the characteristics of the problem domain. Portability allows testing of different systems. Scaleability of the benchmark allows testing systems of different sizes, and simplicity means that the benchmark has to be understandable and thereby credible. Accessibility means that the benchmark has to be publicly available, solvability to set the level of the task on suitable level (not too difficult, not hoo hard), independence to guarantee that the benchmark has not been developed with tools to be tested, and evolution to keep the benchmark up-to-date during time.

When considering the variation benchmarks, datasets should be large enough to cover variations related to a certain feature or mechanism. For example in the case of missense variations this means very large numbers of instances as there are altogether 150 single nucleotide changes which cause amino acid substitution. To have statistical power several cases are needed. The required numbers of cases increase exponentially as features are combined. Datasets have to be non-redundant and devoid of similar or greatly overlapping entries. This criterion relates to independence requirement of [23]. Datasets have to contain both positive (showing the investigated feature) and negative (not having effect) cases so that the capability of methods to distinguish effects can be tested. This may cause problems in data collection as some phenomena are very rare and only a few known cases may exist.

VariBench is a database for variation-related benchmark datasets that can be used for developing, optimizing, comparing and evaluating the performance of computational tools that predict the effects of variations (Nair and Vihinen, submitted). VariBench datasets provide multilevel mapping of the variation position to DNA, RNA and protein as well as to protein structure entries in PDB [24] (when possible). Method developers are requested to submit their datasets to VariBench to be distributed to the community.

VariBench datasets have been collected from literature as well as with data mining approaches from diverse sources. Locus specific databases (LSDBs) are the most reliable source for disease-related data. Although lots of variation data are listed in LSDBs, it would be necessary to capture to databases all the cases from clinical and research laboratories [25,26].

An integral part of databases is the annotation of the entries. For variation information collection it would be extremely important to describe the cases in a systematic and unambiguous way.

Variation Ontology (VariO, http://variationontology.org/) has been developed for systematic description and annotation of variation effects and consequences on DNA, RNA and/or protein including variation type, structure, function, interactions, properties and other features (Vihinen, in preparation). VariO annotated data would allow easy collection of novel dedicated benchmarks.

## Evaluation measures

**Figure 4:**
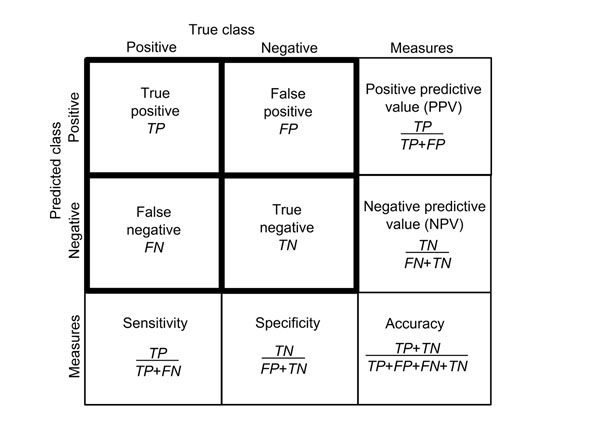
**Contingency matrix and measures calculated based on it** 2x2 contigency table for displaying the outcome of predictions. Based on the table it is possible to calculate row and column wise parameters, PPV and NVP, and sensitivity and specificity, respectively. These parameters are useful, but are not based on all the information in the table. Accuracy is a measure that is calculated based on all the four figures in the table.

The outcome of binary (pathogenic/benign) style predictors are often presented in a 2x2 contingency table (Fig. 4). The number of correctly predicted pathogenic (non-functional) and neutral (functional) cases are indicated by *TP* (true positives) and *TN* (true negatives), and the number of incorrectly predicted pathogenic and neutral cases are *FN* (false negatives) and *FP* (false positives), respectively.

**Figure 5:**
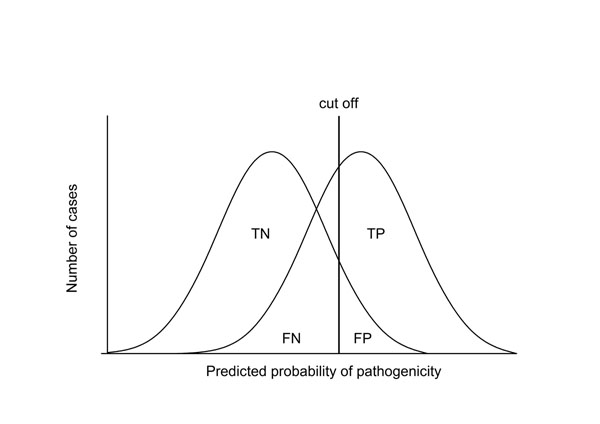
**Separation of classes** In most classification problems the two classes are overlapping. By moving the cut off position the amount of the overlap of the classes can be adjusted. FN and FP are misclassified cases. The prediction methods aim at optimizing the cut off and thereby adjusting the numbers in the contingency table.

The goal of two-class prediction methods is to separate positive cases from negative ones. Because the predictions for the two classes usually overlap a cut off distinguishing the categories has to be optimized (Fig. 5). By moving the cut off different amounts of misclassified cases *FN* and *FP* appear. By using well behaved representative data and well trained classifier the misclassifications can be minimized.

Based on the four descriptors several further measures can be calculated (Fig. 4). Sensitivity, also called true positive rate (*TPR*) or recall, and specificity (true negative rate, *TNR*) show the ratio of the pathogenic and neutral cases correctly identified by the programs. Positive predictive value (*PPV*) (also called precision) and negative predictive value (*NPV*) is the conditional probability that a pathogenic or neutral variant is predicted as pathogenic or neutral, respectively. The mathematical basis of these and other parameters have been discussed in detail [27].

A single parameter cannot capture all the information of the contingency matrix. Unless representative numbers of positive and negative cases are used, the values of NPV and PPV may be biased, even meaningless. The usual requirement is that the numbers be equal. Sometimes in literature the datasets are very skewed. Table 1 indicates the effect of the class imbalance. Results are shown in addition to equally distributed dataset also for analyses when there is ± 25 % or ±50 % difference in the total number of negative and positive cases. In the column wise parameters, which are for the ratios of either positive or negative cases (sensitivity and specificity), are not affected whereas there is a significant difference in NPV and PPV, which are row wise ratios based on numbers of both positive and negative cases. In all the examples, 75 % of both positive and negative cases are correctly predicted and therefore sensitivity and specificity remain the same. It is thus apparent that imbalance in class sizes grossly affects the NPV and PPV evaluation criteria.

**Table 1:** Evaluation measures for test data

	-50 %	-25 %	Equal	+25 %	+50 %
**tp**	750	750	750	750	750
**fn**	250	250	250	250	250
**tn**	375	563	750	938	1125
**fp**	125	187	250	312	375
**sensitivity**	0.75	0.75	0.75	0.75	0.75
**specificity**	0.75	0.75	0.75	0.75	0.75
**PPV**	0.86	0.80	0.75	0.71	0.67
**NPV**	0.60	0.69	0.75	0.79	0.82
**accuracy**	0.75	0.75	0.75	0.75	0.75
**MCC**	0.48	0.50	0.50	0.50	0.49

Example of a situation when both positive and negative cases have the same rate of correct predictions (75 %) and when the amount of negative cases is either equal or 25 or 50 % lower or higher than that for positive cases.

To overcome the class imbalance problem different approaches can be taken. One is to prune the size of the bigger class to be that of the smaller one. It is also possible to normalize in the contingency table the values of either positive or negative cases to have the total of the other class. Quite often in bioinformatics limited amount of data are available and therefore one would be reluctant to delete part of the datasets. When normalizing the data be sure that the existing dataset is representative otherwise bias in the set may further be increased.

### Accuracy and MCC

Specificity, sensitivity, PPV and NPV are calculated by using only half of the information in the contingency table and thus cannot represent all aspects of the performance. Accuracy (Fig. 4) and Matthews correlation coefficient (MMC) take benefit of all the four numbers and as such are more balanced, representative and comprehensive than the line or column wise measures.

The MCC is calculated as follows:

For all the measures discussed in here applies that higher the value the better. Except for MCC, the values range from 0 to 1. MCC ranges from -1 to 1. -1 indicates perfect negative correlation, 0 random distribution and 1 perfect correlation. Accuracy and MCC are affected by class imbalance only in extreme cases.

**Figure 6:**
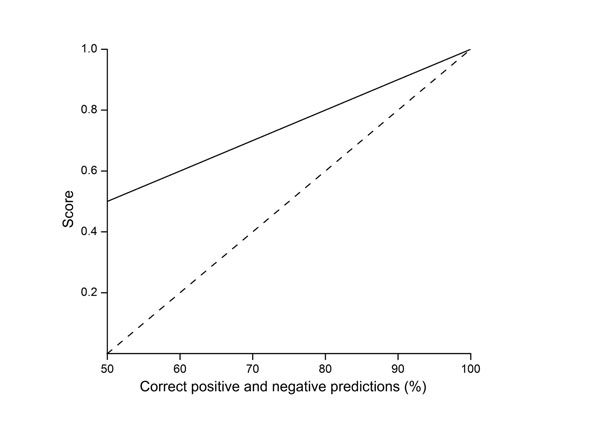
**The growth of the performance measures along increasing reliability** Graphs for quality measures for equally distributed data (same amount positive and negative cases) when the performance increases equally in both classes. The solid curve indicates the growth of sensitivity, specificity, PPV, NPV, and accuracy. The dotted line is for MCC.

The effect of the correctly predicted cases on the parameters in equally distributed dataset is shown in Fig. 6. The value for MCC grows slower than the others reaching 0.5 when 75 % of cases are correctly predicted. Random results (50 % of both negative and positive correctly predicted) gives a value of 0, while the other parameters - sensitivity, specificity, PPV, NPV, and accuracy are 0.5. Fig. 6. can be used to check the performance of equally distributed dataset if e.g. some parameters in an article are not provided. Biases can easily be seen as deviations from the relationships in the figure. To obtain full picture of the predictor performance it is important to evaluate all the six measures together.

### Other parameters

Several other parameters can be derived from the contingency matrix. These are not discussed further as they are not widely used in literature and can be easily calculated from the six previously presented parameters. These include false positive rate (FPR) which equals 1-specificity and false negative rate (FNR) which is 1-sensitivity. False discovery rate (FDR) is 1-PPV.

Positive and negative likelihood ratios are calculated as follows:

F measure is another one that uses all the data. It is calculated as:

Other measures include e.g. Hamming distance and quadratic distance (also called for Euclidean distance), which are the same for binary data, and relative entropy and mutual information [27].

### ROC analysis

Receiver operating characteristics (ROC) analysis is a visualization of prediction performance, that can be used to select suitable classifier (for review see [28,29]). It indicates the tradeoffs between sensitivity and specificity. ROC curves can be drawn with specific programs when the predictor is of probabilistic type and provides a score for the classification. The score is usually not a real p value, but a value usable for ranking the predictions.

**Figure 7:**
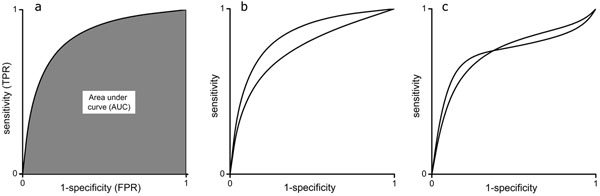
**ROC analysis and AUC** a) Principle of ROC analysis. b) Comparison of predictors based on the ROC curves when the methods are tested with the same dataset (benchmark). c) If the curves cross the comparison is no more meaningful.

ROC curve (Fig. 7a) is drawn by first ranking the data based on the prediction score. Then the data are divided to intervals of equal size. The upper limit for the partitions is the number of cases in the dataset. ROC curve has on x-axis 1-specificity also called FPR and on the y-axis sensitivity (TPR).

Computer program establishes cut offs at intervals, calculates contingency table for data in the interval, and determines the values for sensitivity and 1-specificity, which is plotted to the graph. The procedure is repeated for each partition. If cross validation has been used, then the ROC curve can be used to show the average and variance of the results.

In an ideal case all the true positive cases are on the first half of the ranked list and the plot rises to (0,1) and then continues straight to the right with all the true negative cases. A random classification would be on the diagonal i.e. mixed correct and wrong cases. The faster the curve rises and the higher it reaches in the beginning the better the method is. Methods can be compared with ROC analysis when the same test dataset (benchmark) is used (Fig 7b). The curve that runs higher is for a better method. If the curves cross (Fig 7c) the comparison is no more meaningful.

Area under the ROC curve (AUC) has been used as a measure of goodness for predictions (Fig. 7a). It approximates the probability of ranking a randomly chosen positive instance higher than a randomly chosen negative one. A value of 0.5 indicates random and useless classification while 1 would indicate perfect classifier. Note that AUC can be even smaller than 0.5. One should bear in mind that the ROC curve does not directly indicate the performance of a method. It shows the method’s ranking potential, which is related to overall performance, further strengthening the fact that a single measure cannot fully describe the predictive performance even if it produces a graph.

## What if the data is classified to more than two classes?

If there are more than two classes the measures described above cannot be applied. The data can still be presented in an *N x N* contingency table. One approach is to divide the data into several partitions of two categories.

If parameters are needed for all the classes there are some options available, however, single measures are more problematic. It is possible to calculate row and column wise ratios in the same way as in Fig. 4. MCC is in fact a special case for binary data of linear correlation coefficient, which can be used for several classes in its general format. Mutual information analysis can be used in these cases, as well. Applicable measures have been discussed e.g. in [27].

## Examples of performance comparisons

This section discusses examples of variation effect prediction method evaluations. These include methods for amino acid substitution (missense variation) tolerance, point variation effects on protein stability and variations related to mRNA splicing. The discussion concentrates on the comparison principles, especially in the light of the discussion on requirements mentioned above. The actual comparisons are not presented as it would have required publication of substantial parts of the reports. As a single parameter is insufficient for ranking methods, the readers are directed to the original articles to find all the details. Here a summary to the methodology and use of the evaluation parameters is provided.

### Protein tolerance predictors

Single nucleotide alterations are the most common genetic variation type. Human genomes contain these variations on average at every kilobase. Several computational methods have been developed to classify these variations [1]. The evaluated methods were MutPred, nsSNPAnalyzer, Panther, PhD-SNP, PolyPhen, PolyPhen2, SIFT, SNAP, and SNPs&GO [5]. The methods differ in the properties of the variant they take into account, as well as in the nature and the classification method. Panther, PhD-SNP and SIFT are based on evolutionary information. MutPred, nsSNPAnalyzer, PolyPhen2, SNAP and SNP&GO combine protein structural and/or functional parameters and sequence analysis derived information. Most of these are based on machine-learning methods.

The positive test dataset included 19,335 missense variations from the PhenCode database [30], IDbases [31] and from 18 additional LSDBs. The negative dataset consisted of 21,170 nonsynonymous coding SNPs with an allele frequency >0.01 and chromosome sample count higher than 49 from the dbSNP database. As large numbers of individual predictions were the Pathogenic-or-not Pipeline (PON-P) [32] was used for the submission of sequences and variants into the analysed programs.

The performance was evaluated with the six measures described above. The performances of the programs ranged from poor (MCC 0.19) to reasonably good (MCC 0.65) [5].

It has been widely accepted that information about protein three dimensional structure would increase prediction performance. The very best methods use also structural and functional information, whereas others that are solely based on sequence level information perform rather well.

Further analyses were made to compare the methods pairwise, and to study whether the type of original or substituting amino acid residue, the structural class of the protein, or the structural environment of the amino acid substitution, had an effect on the prediction performance.

Existing programs thus have widely varying performance and there is still need for better methods. Considering all the evaluation measures, no single method could be rated as best by all of them.

### Protein stability predictors

Stability as a fundamental property affects protein function, activity, and regulation. Changes to stability are often found to be involved in diseases. Systematic performance evaluation analysis has been made for eleven stability predictors performances including CUPSAT, Dmutant, FoldX, I-Mutant2.0, two versions of I-Mutant3.0 (sequence and structure versions), MultiMutate, MUpro, SCide, Scpred, and SRide [2]. SCide and Scpred, which predict stability centers, as well as SRide, which predicts stabilizing residues, predict only destabilizing effects, while all the others evaluate both stabilizing and destabilizing changes.

The major database for protein stability information is ProTherm [33]. The pruned dataset for testing contained 1784 variations from 80 proteins, with 1154 positive cases of which 931 were destabilizing (ΔΔG ≥ 0.5 kcal/mol), 222 were stabilizing (ΔΔG ≤ –0.5 kcal/mol), and 631 were neutral (0.5 kcal/mol ≥ ΔΔG ≥ –0.5 kcal/mol). The majority of the methods had been trained using data from ProTherm, and thus only those cases that had been added to the database after training had occurred were used for testing.

Of the measures recommended in here the authors used four, namely accuracy, specificity, sensitivity, and MCC and the remaining row wise parameters could be calculated from the confusion tables.

There were three groups of data, stability increasing, neutral and stability decreasing. The authors solved the problem of multiple classes by presenting three tables of results. The first one was grouped so that both stability increasing and decreasing were considered as pathogenic i.e. positive. In these analyses only two classes were considered, stabilizing or destabilizing and neutral cases.

The results for the all the cases show that accuracy ranges from 0.37 to 0.64 and MCC from -0.37 to only 0.12. All the programs succeeded better when predicting stability increasing or decreasing variations individually. The MCC reaches 0.35 and 0.38 for the methods best in predicting stability increasing and decreasing variants, respectively [2].

Further analyses were made about variations located in different protein secondary structural elements, on the surface or in the core of a protein, and according to protein structure type.

The conclusion was that even at best, the predictions were only moderately accurate (~60%) and significant improvements would be needed. The correlation of the methods was poor.

In another study six programs includeing CC/PBSA, EGAD, FoldX, I-Mutant2.0, Rosetta, and Hunter were compared [3]. The dataset contained 2156 single variations from ProTherm. The goal of the study was to compare the performance of the methods in ΔΔG prediction. Thus, they did not directly predict the effect on protein function, just the extent of free energy change. The only measure used was correlation between the experimental and predicted ΔΔG values.

The ability of Dmutant, two versions of I-Mutant 2.0, MUpro, and PoPMuSiC to detect folding nuclei affected by variations has been evaluated [34]. The dataset contained 1409 variations from the ProTherm and some methods were tested with the same data which they had been trained. They used only correlation coefficients as quality measures. The best being in the range of ~0.5.

The performance of structure-based stability preditors, Dmutant, FoldX, and I-Mutant 2.0, were investigated with data for two proteins. There were 279 rhodopsin and 54 bacteriorhodopsin variations [35]. The best prediction accuracy for the rhodopsin dataset was <0.60, while it was somewhat greater for the bacteriorhodopsin dataset.

### Splice site predictors

mRNA maturation is a complex process, which may be affected by variations in many steps. Prediction behaviour of nine systems, GenScan, GeneSplicer, Human Splicing Finder (HSF), MaxEntScan, NNSplice, SplicePort, SplicePredictor, SpliceView and Sroogle was tested [4].

The test dataset contained altogether 623 variations. The first dataset contained 72 variations that affect the four invariant positions of 5’ and 3’ splice sites. The second one included 178 variations either localized at splice sites in non-canonical positions, distant intronic variations, and short distance variations. The third set of 288 exonic variations included 10 exonic substitutions that activate a cryptic splice site. In the fourth dataset were negative controls, altogether 85 variations without effect on splicing.

The results contain just the numbers of predicted cases and the percentage of correct ones, thus detailed analysis of the merits of the methods cannot be made.

The authors recommended some programs but stated that the *in silico* predictions need to be validated *in vitro*.

## Checklist for method developers and users

This checklist is provided to help when comparing and measuring performance of predictors and when selecting a suitable one. These are items that method developers should include in articles, or as supplement to articles, as they enable effective comparison and evaluation of the performance of predictors.

Items to check when estimating method performance and comparing performance of different methods:

- Is the method described in detail?

- Have the developers used established databases and benchmarks for training and testing (if available)?

- If not, are the datasets available?

- Is the version of the method mentioned (if several versions exist)?

- Is the contingency table available?

- Have the developers reported all the six performance measures: sensitivity, specificity, positive predictive value, negative predictive value, accuracy and Matthews correlation coefficient. If not, can they be calculated from figures provided by developers?

- Has cross validation or some other partitioning method been used in method testing?

- Are the training and test sets disjoint?

- Are the results in balance e.g. between sensitivity and specificity?

- Has the ROC curve been drawn based on the entire test set?

- Inspect the ROC curve and AUC.

- How does the method compare to others in all the measures?

- Does the method provide probabilities for predictions?

## Competing interests

The author declares that they have no competing interests in relation to the SNP-SIG issue article.
